# Efficacy of Microneedle Fractional Radiofrequency Combined With Topical Insulin for the Treatment of Facial Atrophic Acne Scars: A Split‐Face, Double‐Blinded, Randomized, Placebo‐Controlled Trial

**DOI:** 10.1111/jocd.70033

**Published:** 2025-02-19

**Authors:** Teerapong Rattananukrom, Kasama Tejapira, Cherrin Pomsoong, Yanisa Ratanapokasatit, Vasanop Vachiramon

**Affiliations:** ^1^ Division of Dermatology Faculty of Medicine Ramathibodi Hospital, Mahidol University Bangkok Thailand

**Keywords:** atrophic acne scar, collagen synthesis, microneedle fractional radiofrequency, topical insulin

## Abstract

**Introduction:**

Topical insulin (TI) has been shown to enhance wound healing by promoting re‐epithelialization and collagen synthesis. However, there have been limited studies addressing its potential use in treating acne scars.

**Objectives:**

To assess the efficacy of combining TI with microneedle fractional radiofrequency (MFR) in the treatment of atrophic acne scars.

**Materials and Method:**

A split‐face, double‐blinded, placebo‐controlled trial involved 30 participants with atrophic acne scars. Each side of the participants' face was randomly treated with a combination of MFR and TI or a placebo every 4 weeks for four consecutive sessions.

**Results:**

Significant improvements in scar volume were observed, with statistical significance at week 12 for MFR combined with TI sides (baseline 18.22 ± 9.86 vs. 16.20 ± 8.58 mm^3^, *p* = 0.017) and at week 16 for MFR combined with placebo sides (baseline 18.02 ± 9.24 vs. 15.28 ± 8.21 mm^3^, *p* = 0.001). The TI sides exhibited a reduction of 46.77% ± 21.33% in Echelle d’ évaluation clinique des cicatrices d’ acne (ECCA) (*p* ≤ 0.001), while the placebo sides showed a reduction of 46.39% ± 20.14% in ECCA (*p* ≤ 0.001). There were no significant differences in melanin, and hemoglobin index between the two groups. No hypoglycemic symptoms were reported.

**Conclusion:**

Combining topical insulin with MFR is safe and could accelerate the early acne scar improvement.

## 1 Introduction

Atrophic acne scars represent the most common sequelae of inflammatory acne, affecting approximately 90% of adults [1, 2]. These scars can significantly impact patients' cosmetic concerns and self‐confidence [3]. The likelihood of developing acne scars is influenced by the intensity and duration of the inflammatory process and an imbalance in matrix metalloproteinases (MMPs) to tissue inhibitors of MMPs during extracellular matrix remodeling. This imbalance weakens collagen synthesis and contributes to the formation of atrophic scars [4]. Among acne scars, over 80% are atrophic in nature, primarily comprised of icepick scars (60%), followed by boxcar scars (25%) and rolling scars (15%) [4].

One innovative treatment approach for atrophic acne scars is Microneedle Fractional Radiofrequency (MFR), which utilizes an array of electrodes to deliver electrical currents to specific depths of the dermis. This process induces controlled thermal injury, thereby stimulating collagen production [5, 6]. Numerous studies have explored the combination of MFR with adjuvant topical treatments to improve drug delivery and enhance the treatment of atrophic acne scars.

Insulin, a crucial peptide hormone, plays a pivotal role in the wound‐healing process [7]. Topical insulin (TI) has been shown to promote wound healing through multiple pathways, including the enhancement of collagen synthesis [8, 9, 10, 11]. This property makes it a promising candidate for combination treatment in atrophic acne scars, with the potential to restore collagen in the extracellular matrix.

This study was conducted to assess the efficacy of TI in combination with MFR for the treatment of atrophic acne scars.

## 2 Materials and Methods

### 2.1 Population and Study Design

This single‐center study was a randomized, split‐face, double‐blinded, placebo‐controlled trial conducted at Ramathibodi Hospital, Mahidol University, Bangkok, Thailand, between November 2021 and August 2022, in compliance with the Declaration of Helsinki. The study protocol received approval from the Mahidol Institutional Review Board for Human Subject Research (COA. MURA2021/727), Faculty of Medicine, Ramathibodi Hospital, Mahidol University, Bangkok, Thailand. The trial was registered with the Thai Clinical Trial Registry (TCTR20211203003). All patients provided written informed consent before enrolling in the study.

Thirty healthy participants aged 18–50 years, with atrophic acne scars on both cheeks were eligible. Participants were excluded if they met any of the following criteria: pregnancy or breastfeeding, use of immunosuppressive drugs, prior laser treatment for acne scars within the last 3 months, use of chemical peeling within the last 3 months, use of systemic retinoids within the last 3 months, allergy to insulin or xylocaine, history of photosensitivity, history of skin cancer, history of diabetes mellitus, history of connective tissue disease, history of keloid or hypertrophic scars at any site, and inability to comply with the follow‐up protocol.

A block randomization scheme with a block size of two was employed to determine which side of each participant's face would receive either TI or a placebo in conjunction with MFR. A 5% lidocaine‐prilocaine cream (RacSER) was applied to both sides of the patients' faces under occlusion for a duration of 60 min. The RacSER cream was thoroughly washed off before treatment to prevent interference with the absorption of TI. Subsequently, MFR using the Fractora device (Inmode Aesthetics, Lake Forest, CA) was administered. The treatment utilized a tip equipped with 60 pins, delivering energy in the range of 15–30 mJ/pin, with 1–2 passes performed for all participants until achieving mild to moderate erythema with some pinpoint bleeding on acne scars. Air cooling was employed during and after the MFR procedure to ensure participant comfort and facilitate skin cooling. Following MFR, patients received either 2 mL of TI (Human Actrapid Insulin, 40 IU/mL solution, NovoNordisk India Pvt. Ltd., Bangalore, India) or a normal saline solution (NSS) under occlusion for a duration of 30 min. Patients were provided with specific instructions to apply fucidic acid ointment twice daily for a period of 14 days, to avoid sun exposure, and to apply sunscreen daily. Each participant underwent a total of four treatment sessions at baseline, week 4, week 8, and week 12. All participants were blinded to the topical treatment on each side of their faces. Additionally, the investigators were blinded to the treatment protocol and evaluated the outcomes based on clinical photographs.

### 2.2 Study Outcomes

The primary outcome of this study was the evaluation of atrophic acne scar improvement at week 16 and 24 compared to the baseline. This assessment involved the objective measurement of scar volume, skin roughness, melanin index, and hemoglobin index using a standardized 3D camera system (Antera 3D, Miravex, Ireland) at a consistent reference point for each participant during every follow‐up visit. Additionally, clinical photographs were captured from both the frontal and lateral aspects of each hemiface using the Visia skin analysis imaging system (Visia CR, Canfield Imaging Systems, Farfield, NJ, USA) during each follow‐up visit. Clinical assessment, employing the Echelle d’ évaluation clinique des cicatrices d’ acne (ECCA) grading scale, was conducted by two blinded dermatologists at the baseline, week 16, and week 24. The secondary outcome measures included skin roughness, melanin index, hemoglobin index, as well as participants' self‐assessment of pain, improvement, and self‐satisfaction using a visual analog scale (VAS, 0–10 cm).

### 2.3 Statistical Analysis

Baseline characteristics were summarized using descriptive statistics, presented as either mean and standard deviation or median and interquartile range. The improvement in scar volume, skin roughness, melanin, and hemoglobin index, as well as the ECCA score, was analyzed using a linear mixed‐effect model. Comparison of VAS scores was conducted using a paired *t*‐test. All statistical analyses were performed using the STATA program (STATA/SE version 14, STATA Corp, College Station, TX). Statistical significance was defined as *p* ≤ 0.05.

## 3 Results

### 3.1 Subject Demographics

A total of thirty participants with atrophic acne scars were enrolled in the study. The demographic characteristics are summarized in Table 1. The mean age of the participants was 30.66 ± 6.74 years, with a male‐to‐female ratio of 16:13. Baseline comparisons revealed no statistically significant differences between the two treatment groups regarding scar volume, skin roughness, melanin levels, hemoglobin index, and ECCA scores. Prior to the initiation of the intervention, one participant dropped out due to an inability to comply with the follow‐up protocol. Consequently, the study was completed with a total of 29 subjects who participated in the follow‐up assessments.

**TABLE 1:** Demographic and baseline characteristics.

Characteristics	MFR and topical insulin	MFR and placebo	*p*
Age, mean ± SD		30.66 ± 6.74	
Gender			
Male, *n* (%)		13 (44.83%)	
Female, *n* (%)		16 (55.17%)	
Fitzpatrick skin type			
Type 3, *n* (%)		11 (37.93%)	
Type 4, *n* (%)		18 (62.07%)	
Atrophic scar volume (mm^3^), mean ± SD	18.22 ± 9.86	18.02 ± 9.24	0.814
Skin roughness, mean ± SD	24.93 ± 7.86	24.93 ± 7.44	0.992
Echelle d’ évaluation clinique des cicatrices d’ acne (ECCA) grading scale			
Total, mean ± SD	156.21 ± 39.57	153.79 ± 41.37	0.658
V type, mean ± SD	39.31 ± 8.42	40.34 ± 8.12	0.535
U type, mean ± SD	49.65 ± 13.75	49.66 ± 13.75	1.00
M type, mean ± SD	62.07 ± 15.84	58.62 ± 16.74	0.344
Melanin index, mean ± SD	0.60 ± 0.07	0.61 ± 0.07	0.967
Hemoglobin index, mean ± SD	1.43 ± 0.16	1.44 ± 0.16	0.683

Abbreviation: MFR, microneedle fractional radiofrequency.

### 3.2 Echelle d’ évaluation Clinique Des Cicatrices d’ Acne (ECCA) Grading Scale

Both treatment groups demonstrated a significant improvement in ECCA scores after completing the four treatment sessions. The MFR combined with TI sides exhibited a reduction of 46.77% ± 21.33% in ECCA scores (from a baseline of 156.21 ± 39.57 to 84.66 ± 42.57; *p* ≤ 0.001), while the MFR combined with placebo sides showed a reduction of 46.39% ± 20.14% (from a baseline of 153.79 ± 41.37 to 83.79 ± 42.10; *p* ≤ 0.001) in Table 2. There was no statistically significant difference in ECCA score reduction between the two treatment groups at the last visit (*p* = 0.728). Significant reductions in ECCA scores were observed across all subtypes (*p* ≤ 0.001 for all subtypes) with no statistically significant differences between the treatments for each subtype. [V type (icepick scar), *p* = 0.207; U type (rolling scar), *p* = 0.251; M type (boxcar scar), *p* = 0.541].

**TABLE 2:** échelle d'évaluation clinique des cicatrices d'acné (ECCA) score.

Physician evaluation	MFR and TI	MFR and placebo	*p* (between group)
Total ECCA			0.728
Baseline, mean ± SD	156.21 ± 39.57	153.79 ± 41.37	
Week 24 after treatment, mean ± SD	84.66 ± 42.57	83.79 ± 42.10	
*P*‐value (within group)	*p* ≤ 0.001^a^	*p* ≤ 0.001^a^	
V type (icepick scar)			0.207
Baseline, mean ± SD	39.31 ± 8.42	40.34 ± 8.12	
Week 24 after treatment, mean ± SD	23.28 ± 7.60	25.34 ± 7.80	
*P*‐value (within group)	*p* ≤ 0.001^a^	*p* ≤ 0.001^a^	
U type (rolling scar)			0.251
Baseline, mean ± SD	49.65 ± 13.75	49.66 ± 13.75	
Week 24 after treatment, mean ± SD	30.34 ± 13.08	27.59 ± 12.70	
*P*‐value (within group)	*p* ≤ 0.001^a^	*p* ≤ 0.001^a^	
M type (boxcar scar)			0.541
Baseline, mean ± SD	62.07 ± 15.84	58.62 ± 16.74	
Week 24 after treatment, mean ± SD	25.00 ± 13.51	26.72 ± 13.02	
*P*‐value (within group)	*p* ≤ 0.001^a^	*p* ≤ 0.001^a^	

Abbreviations: MFR, microneedle fractional radiofrequency; TI, topical insulin.

^a^ Described as the statistical significance.

### 3.3 Three‐Dimensional Image Analysis Measured Using Antera3D

#### 3.3.1 Atrophic Acne Scar Volume

**FIGURE 1:**
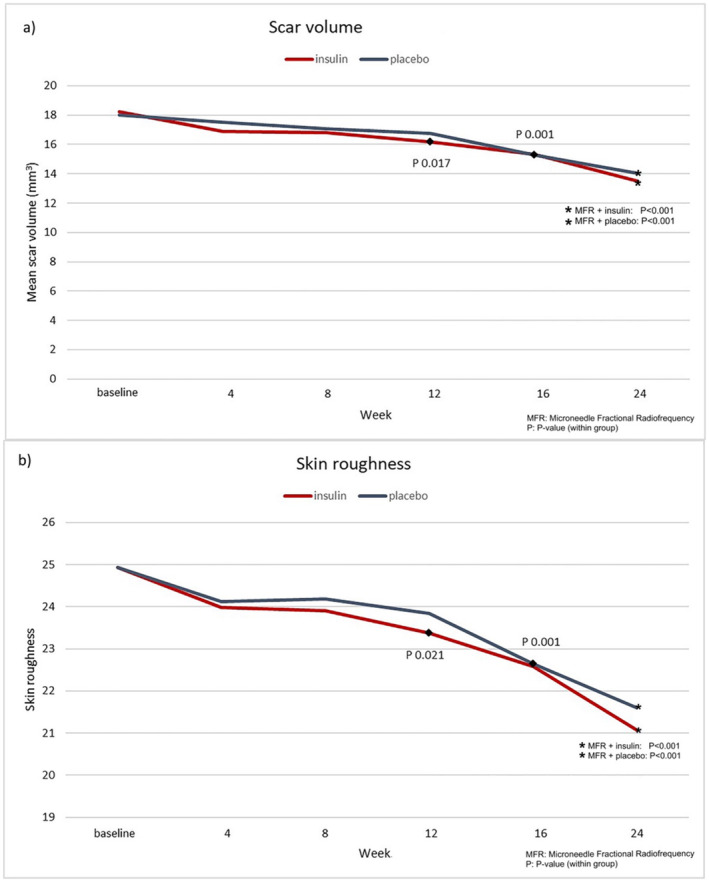
Both treatments showed significant improvements in atrophic acne scar volume (a) and skin roughness (b) at week 12 for insulin‐treated sides and at week 16 for placebo‐treated sides.

**FIGURE 2:**
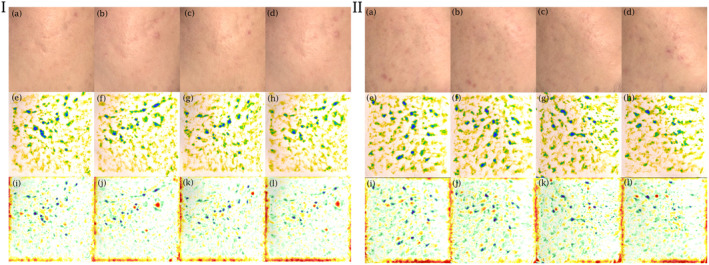
Clinical photographs and 3‐D images illustrating improvement in scar volume (e–h) and skin roughness (i–l) in a participant who underwent MFR combined with insulin on the left (I) and MFR combined with placebo on the right (II) cheek, from baseline (a, e, i) to week 12 (b, f, j), week 16 (c, g, k), and week 24 (d, h, l) of treatment.

Significant improvements in atrophic acne scar volume were observed, with statistical significance at week 12 for TI‐treated sides (baseline 18.22 ± 9.86 vs. 16.20 ± 8.58 mm^3^, *p* = 0.017) and at week 16 for placebo‐treated sides (baseline 18.02 ± 9.24 vs. 15.28 ± 8.21 mm^3^, *p* = 0.001) compared to baseline (Figure 1a). In addition, at the last follow‐up, acne scar volume shown significant improvements in both groups compared to baseline [(TI: 24th week 13.48 ± 6.97 mm^3^, *p* < 0.001) (placebo: 24th week 14.03 ± 7.53 mm^3^, p < 0.001)]. However, no significant difference in scar volume improvement was observed between TI and placebo‐treated sides after treatment completion (*p* = 0.517) as shown in Figure 2 and Table 3.

**TABLE 3:** Analysis of atrophic acne scar volume and skin roughness at week 12, 16, and 24 after treatment, evaluated using a 3‐Dimensional imaging system.

3‐Dimensional imaging system	MFR and topical insulin	*p* (within group)	MFR and placebo	*p* (within group)	*p* (between group)
Atrophic acne scar volume, mean ± SD					
Baseline	18.22 ± 9.86		18.02 ± 9.24		0.814
Week 12 after treatment	16.20 ± 8.58	0.017^a^	16.75 ± 8.63	0.132	0.518
Week 16 after treatment	15.31 ± 8.48	0.001^a^	15.28 ± 8.21	0.001^a^	0.967
Week 24 after treatment	13.48 ± 6.97	< 0.001^a^	14.03 ± 7.53^a^	< 0.001^a^	0.517
Skin roughness, mean ± SD					
Baseline	24.93 ± 7.86		24.93 ± 7.44		0.992
Week 12 after treatment	23.38 ± 6.74	0.021^a^	23.85 ± 7.33	0.112	0.479
Week 16 after treatment	22.58 ± 6.20	0.001^a^	22.65 ± 6.71	0.001^a^	0.926
Week 24 after treatment	21.07 ± 5.57	< 0.001^a^	21.60 ± 6.11	< 0.001^a^	0.429

Abbreviation: MFR, microneedle fractional radiofrequency.

^a^ Described as the statistical significance.

#### 3.3.2 Skin Roughness

Similarly, skin roughness demonstrated significant improvement at week 12 for TI‐treated sides (baseline 24.93 ± 7.86 vs. 23.38 ± 6.74, *p* = 0.021) and at week 16 for placebo‐treated sides (baseline 24.93 ± 7.44 vs. 22.65 ± 6.71, *p* = 0.001) when compared to baseline (Figure 1b). Image analysis indicated slightly greater improvement on the TI‐treated sides (24th week 21.07 ± 5.57, *p* < 0.001) in comparison to the placebo‐treated sides (24th week 21.60 ± 6.11, *p* < 0.001). However, there was no statistically significant difference between the two treatments at the end of the study (*p* = 0.429) as shown in Figure 2 and Table 3.

#### 3.3.3 Melanin and Hemoglobin Index

The melanin index, reflecting post‐inflammatory hyperpigmentation, exhibited slight increases at the end of the study in both treatment groups. However, the increase was not different between the two treatment arms (*p* = 0.504). Hemoglobin index after treatment completion was comparable to baseline in both arms.

#### 3.3.4 Patients' Self‐Evaluation and Satisfaction

**FIGURE 3:**
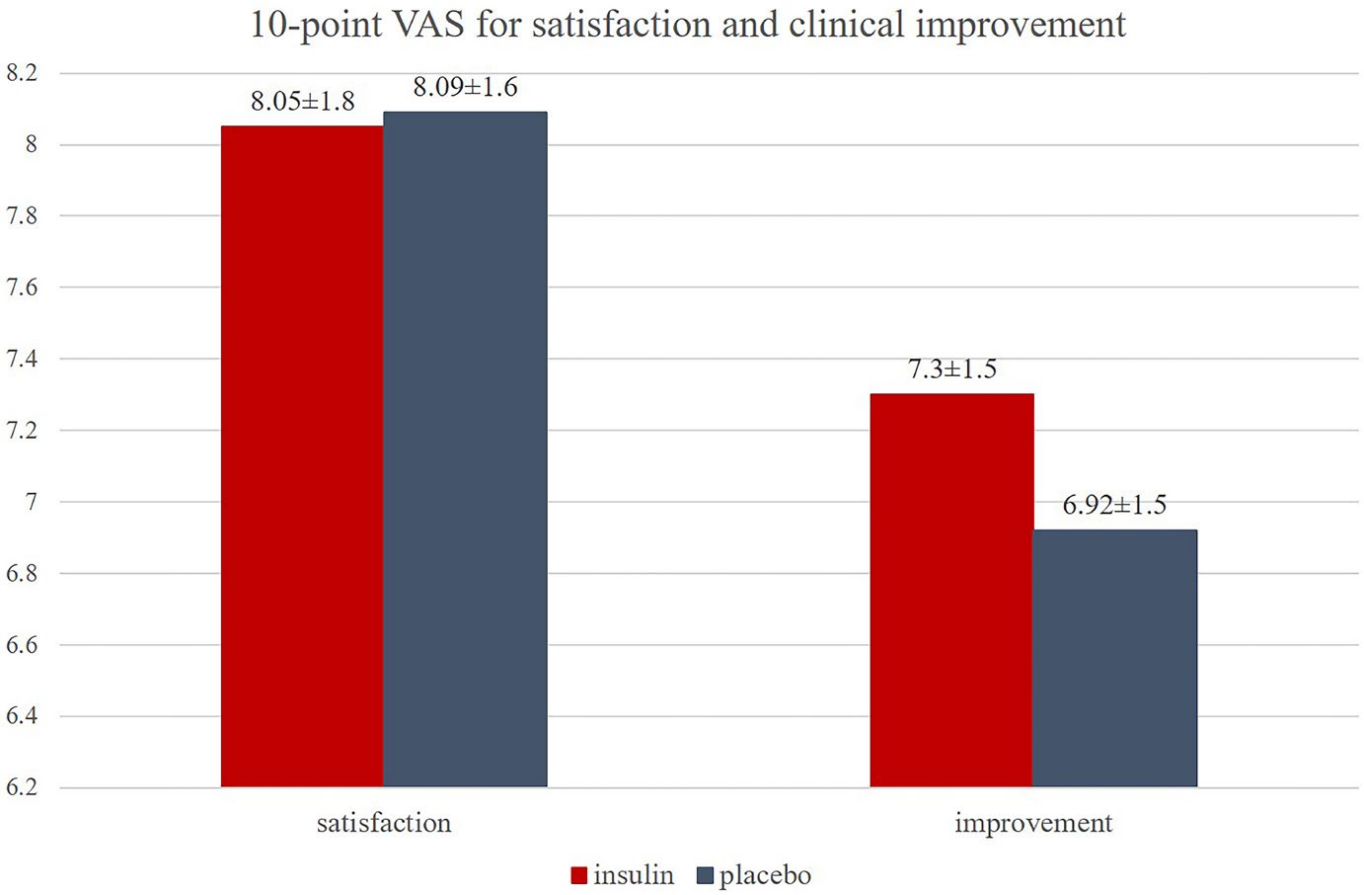
Clinical efficacies of both treatments are demonstrated as improvements and satisfaction on a patient's self‐ rated 10‐point visual analogue scale.

At the last follow‐up, participants assessed their self‐rated 10‐point Visual Analogue Scale (VAS) for scar improvement and satisfaction (Figure 3). Self‐rated improvement scores for both treatment groups were also comparable (TI: 7.30 ± 1.50 vs. placebo: 6.92 ± 1.50, *p* = 0.37). The VAS scores for patients' satisfaction were 8.05 ± 1.80 for the TI‐treated sides and 8.09 ± 1.60 for the placebo‐treated sides (*p* = 0.944).

#### 3.3.5 Adverse Effects

No hypoglycemic symptoms were reported during the study. Participants experienced only temporary pain during treatment, which was well‐tolerated. This pain consistently decreased at each visit. Post‐treatment erythema following MFR resolved within a few days without requiring further intervention. One patient experienced post‐inflammatory hyperpigmentation on both sides of their face, and no significant difference in adverse effects was observed between the two sides of the face.

## 4 Discussion

To the best of our knowledge, this study represents the first split‐face, double‐blinded, randomized, placebo‐controlled trial conducted in the Asian population, comparing the efficacy of MFR combined with TI or placebo in the treatment of facial atrophic acne scars. Our findings suggest that the combination of TI with MFR led to statistically significant improvements in scar volume and skin roughness by week 12 of treatment. In contrast, these significant improvements in the placebo‐treated halves was observed at week 16. However, at the last follow‐up, both interventions demonstrated statistically significant improvements in atrophic acne scars. These findings were corroborated by dermatologist assessments using the ECCA scoring system, as well as patient self‐evaluation and satisfaction scores. Importantly, no statistically significant differences were observed in the degree of improvement between the two interventions. Adverse effects, including transient pain, erythema, and post‐inflammatory hyperpigmentation, were well‐tolerated in both groups, and no hypoglycemic symptoms were reported.

To date, only two previous studies have investigated the efficacy of TI in the treatment of atrophic acne scars. In the study by Pawar et al. [12], a split‐face trial involving 16 patients was conducted to compare the efficacy of micro‐needling with TI (Human Actrapid Insulin, 40 IU/L solution; Novo Nordisk India Pvt. Ltd., Bangalore, India) or autologous platelet‐rich plasma (PRP) on different halves of the patients' faces. The treatment involved three sessions at monthly intervals. The findings revealed that the use of TI on one side of the face led to a comparable outcome to the application of autologous PRP in terms of reducing atrophic acne scar volume. Specifically, the side treated with TI exhibited a 45% improvement, while the PRP‐treated side showed a 26% improvement in atrophic scars. In another study conducted by Abbas et al. [13], a split‐face trial consisting of 30 patients compared the use of TI (Human Actrapid Insulin) versus the application of 17% topical vitamin C following micro‐needling. The treatment consisted of four sessions, each administered at monthly intervals. The study illustrated a slight improvement in the acne scar assessment scale in the vitamin C‐treated sides.

Although a direct comparison with previous studies cannot be performed due to the use of different interventions and evaluation parameters, the results from both studies demonstrated that adjuvant TI with micro‐needling proved to be an effective modality in the treatment of acne scars. In addition, MFR or micro‐needling enhances topical cutaneous delivery of drugs to specific depths of the dermis and accelerates a more favorable response in acne scar treatment.

Focusing on MFR creates a zone of coagulative necrosis, stimulating the release of growth factors and cytokines and also sparing adnexal structures and the epidermis, leading to rapid healing [14]. Typically, improvements in acne scar appearance are observed around 12 weeks after the completion of 3–4 treatment sessions. This explanation can be attributed to the adequate activation of fibroblasts and upregulation of collagen production in the dermal matrix [6]. Furthermore, it has been reported that MFR improves atrophic acne scars by inducing transforming growth factor‐β (TGF‐β) expression within 2 days after the intervention [15], subsequently activating dermal fibroblasts and enhancing collagen formation in a concentration‐dependent manner [5, 16].

Regarding TI and its effect on acne scars, it has been demonstrated that TI enhances keratinocyte migration, accelerates re‐epithelialization, and increases fibroblastic reaction [8]. TI stimulates the phosphatidylinositol 3‐kinase (PI3K)/protein kinase B (Akt) pathways, which promote the release of vascular endothelial growth factor (VEGF), enhancing the recruitment of endothelial progenitor cells (EPC) to the wound. This, in turn, accelerates vascular formation and re‐epithelialization [17] and supporting extracellular matrix remodeling. Additionally, TI accelerates 3H‐thymidine incorporation, indicating cellular mitotic division activity [18], in human skin fibroblasts [19, 20]. Insulin promotes collagen production in a normal skin‐like organization, as opposed to the crosslinking and parallel alignment observed in scar tissue [21]. Intriguingly, insulin‐like growth factor (IGF‐1), which shares homologous sequences with insulin [22], activates fibroblasts via TGF‐β, albeit in a transient manner [23]. Therefore, TI is believed to activate collagen production in fibroblasts through the TGF‐β pathway.

We hypothesize that the immediate application of TI, in addition to the physiological upregulation by day 2 following MFR, may initiate the collagen synthesis pathway earlier, leading to a more rapid reduction in scar volume in the TI‐treated areas. However, the exact pathogenesis of TI combined with MFR in improving acne scars remains unknown. Further research into the cytokine release mechanisms involved in the combination of these modalities should be investigated.

However, at the end of our study, the efficacy of TI with MFR was not different from the placebo with MFR in improving atrophic scar volume and skin roughness. This outcome could be explained by the dynamic regeneration of both collagen and elastin, mediated through multiple cytokines and molecular pathways in every single patient [24]. Several factors impact treatment response, including the number of treatment sessions, the stability of insulin applied immediately after MFR on the treated area, which has cumulative heat, and cutaneous absorption of insulin. Moreover, the role of insulin in the healing process and collagen stimulation in healthy, nondiabetic hosts is still unknown. In contrast to diabetic hosts, whose fibroblasts exhibit selective impairments in cellular processes involved in tissue healing, specifically cellular migration, VEGF production, and tissue response to hypoxia [25]. These impairments contribute to reduced collagen production, which could mitigate the effect of topical insulin on collagen synthesis [26]. This phenomenon is evident in an animal model study on the effects of topical insulin on burn wound healing, where topical insulin increased collagen deposition in diabetic rats but had no such effect in nondiabetic subjects [27].

Insulin is typically administered subcutaneously, allowing it to diffuse through the extracellular matrix and be absorbed into nearby capillaries. Actrapid insulin, a short‐acting form, generally begins to work within 30 min of injection, peaks between 1.5 to 3.5 h, and has an action duration of approximately 7–8 h. There is no universal maximum dose for Actrapid insulin; however, the dosage is usually determined by the healthcare provider based on individual needs. For patients using multiple daily injections, the total daily insulin dose often ranges from 0.4 to 1.0 units per kilogram of body weight, divided among the injections [28].

While the exact percentage of absorption can vary, the bioavailability of subcutaneously injected insulin is generally estimated to be between 60%–80%. Specific percentages for dermal absorption are not always provided, but it is assumed to be higher than in the epidermis due to the presence of capillaries, yet lower than subcutaneous absorption, approximately 40%–70%, depending on methods such as MFR or other advanced techniques targeting the dermis.

As a general rule, topical penetration beyond the corneal layer does not occur with molecules larger than 500 Da. Given that insulin weighs over 5000 Da, its size prevents dermal absorption. MFR induces tissue ablation, coagulation, and heating. Histological findings immediately post‐treatment revealed demarcated zones of ablation, coagulation, necrosis, and subnecrosis extending to a depth of 450 μm. Higher energy levels resulted in deeper effects. MFR can potentially enhance the transdermal drug delivery of insulin [29].

Regarding the risk of hypoglycemia due to systemic absorption of topically applied insulin, blood glucose levels measured before and after the procedure showed no significant changes, indicating minimal systemic absorption [12, 17].

Our study had limitations, including a relatively small sample size and the single application of topical insulin per treatment session, despite its short half‐life. The latter factor may have influenced the clinical response in scar improvement. The potential benefits of multiple applications of insulin after MFR until the complete healing process could be further investigated.

## 5 Conclusion

This study demonstrates the first split‐face, double‐blinded, randomized, placebo‐controlled trial comparing TI or placebo in combination with MFR for the treatment of facial atrophic acne scars. Combining topical insulin with MFR is safe and could accelerate the early acne scar improvement. However, the lack of a significant difference between the TI and placebo groups warrants further investigation to elucidate the potential mechanism and additional benefits of TI.

## Author Contributions

Conceptualization: Yanisa Ratanapokasatit, Cherrin Pomsoong, and Teerapong Rattananukrom. Methodology: Teerapong Rattananukrom, Yanisa Ratanapokasatit and Cherrin Pomsoong. Validation: Kasama Tejapira, Vasanop Vachiramon and Teerapong Rattananukrom. Writing‐original draft preparation: Kasama Tejapira and Teerapong Rattananukrom. Critically revising for intellectual content: Teerapong Rattananukrom. Final approval: Teerapong Rattananukrom. All authors have read and agreed to the published version of the manuscript.

## Ethics Statement

The present participants in this project were adhered to all Helsinki ethical principles. The study protocol received approval from the Mahidol Institutional Review Board for Human Subject Research (COA. MURA2021/727), Faculty of Medicine, Ramathibodi Hospital, Mahidol University, Bangkok, Thailand. The trial was registered with the Thai Clinical Trial Registry (TCTR20211203003).

## Conflicts of Interest

The authors declare no conflicts of interest.

## Data Availability

The data that support the findings of this study are available from the corresponding author upon reasonable request.
